# Worse Outcomes Associated With Liver Transplants: An Increasing Trend

**DOI:** 10.7759/cureus.17534

**Published:** 2021-08-29

**Authors:** Nabeel A Siddiqui, Nayaab Ullah, Javeryah R Shaikh, Sanjay Bhandari, Uzma Ullah, Summaya F Khan, Omar Q Khan, Mubeen Khan Mohammed Abdul

**Affiliations:** 1 Research, California Institute of Behavioral Neurosciences & Psychology, Fairfield, USA; 2 Hematology and Oncology, Windsor University School of Medicine, Cayon, KNA; 3 Medicine, Jinnah Sindh Medical University (SMC), Karachi, PAK; 4 Internal Medicine, Medical College of Wisconsin, Milwaukee, USA; 5 Medicine, Loyola University Chicago, Chicago, USA; 6 Medicine, Windsor University School of Medicine, Cayon, KNA; 7 Biology, University of California, Riverside, USA; 8 Hepatology, Aurora St. Luke's Medical Center, Milwaukee, USA

**Keywords:** liver transplantation, national inpatient sample database, mortality, cirrhosis, end-stage liver disease

## Abstract

Background and aim

Since individuals in the early stages of liver cirrhosis are typically asymptomatic, the prevalence of liver cirrhosis may be underestimated. Liver cirrhosis has a significant morbidity and mortality rate, with 1.03 million deaths worldwide each year. For end-stage liver disease, liver transplantation is a potential therapeutic option. The goal of our research was to examine the current trend in liver transplants using data from a national database.

Methods

Using the International Classification of Diseases (ICD)-9 codes, we identified individuals who had a liver transplant during the index hospital admission in the Nationwide Inpatient Sample from 2007 to 2011. This national sample of patients is from the United States. We looked at the yearly trend in liver transplants and related outcomes, such as duration of hospitalization (DOH), hospital expenses, and mortality in the hospital. In order to find determinants of mortality, we used a multivariate analysis.

Results

There were 25,331 patients hospitalized (weighted for national estimate). Between 2007 and 2011, the number of transplants grew by 1.2%. The majority of transplant recipients were Caucasian (57%), with an average age of 54 years, had a private healthcare plan (53%), and had average earnings in the upper quartile by zip code (26%). Patients with a higher Charlson Comorbidity Index (79% had a score of four) were more likely to be admitted to a southern hospital (33%), an academic hospital (>99%), and a large capacity hospital (90%). Seventy percent of liver transplant recipients received cadaver donors. Hepatitis C was the most prevalent reason for transplant (30%), followed by hepatocellular carcinoma (HCC) (29%) and alcoholic liver disease (25%). In 2011, compared to 2007, there was an upward rise in fatality (from 3.8% to 5.1%), average hospital expenditures (from $335,504 to $498,369), and DOH (from 17.4 to 22.7 days). The cost of hospitalization was two billion dollars per year. The independent variables related to an increased mortality on multivariate analysis were African American race (OR: 2.0, 95%, CI: 1.2-3.2; p=0.005) and large capacity hospitals (OR: 2.5, 95% CI: 1.6-4.1; p=0.0002). Predictors linked to lower mortality included private healthcare coverage (vs. Medicare: OR: 0.7, 95%, CI: 0.51-0.97; p=0.03), academic hospital (OR: 0.6, 95% CI: 0.4-0.8; p=0.005), cadaver donor (OR: 0.6, 95% CI: 0.5-0.8; p=0.002), HCC (OR: 0.6, 95% CI: 0.4-0.9; p=0.01), and non-alcoholic steatohepatitis (NASH) cirrhosis (OR: 0.4, 95% CI: 0.2-0.9; p=0.02).

Conclusion

Our study found an increasing trend in worse outcomes (increased mortality, average hospital costs, and average DOH) after a liver transplant. Patients of the African American race and large capacity hospitals were associated with a higher risk of death, whereas private healthcare plans, academic hospitals, cadaver donors, HCC, and NASH cirrhosis were associated with a lower risk.

## Introduction

Orthotropic liver transplantation is considered the only definite treatment available for patients with end-stage liver disease. As of 2017, 14,360 candidates were on the waiting list for a liver transplant for their survival. Since Dr. Starzl performed the first liver transplant in 1963, the process of Deceased Donor Liver (DDL) allocation for recipients evolved for two decades. Before 1997 the liver acquisition allocation was based on hospital status and accumulated time on the waiting list [1]. The patients with acute fulminant liver failure and imminent death could not get liver transplantation just because of a lack of accumulated waiting time.

Modified United Network for Organ Sharing (UNOS) criteria introduced in 1998 relied heavily on the Child-Turcotte-Pugh (CTP) score, stratifying patients according to several clinical and biochemical aspects of liver disease [1]. By incorporating acute clinical aspects like the presence of ascites or hepatic encephalopathy to score patients and better assess the severity of liver dysfunction in the acute setting, the CTP score made it easier to allocate the liver to acute fulminant liver failure patients.

The problem of stratification still persisted in the chronic liver disease group. As a response to the United States government's mandate of "final rule" to deemphasize waiting time in 2002, the Model for End-Stage Liver Disease (MELD) was introduced and, for the first time in decades, the number of patients on the waiting list has decreased [1,2]. In addition, the rates of transplants in African Americans and Asians significantly increased in the MELD era. However, data has been minimal in recent years regarding mortality outcomes post-liver transplantation in hospitalized patients in the MELD; moreover, the MELD score is less accurate in predicting post-transplant mortality outcomes.

Socioeconomic status and other comorbidities have been factored into the new liver allocation policy, making liver transplantation a viable option in elderly people with additional comorbidities [3]. Few studies have attempted to evaluate these effects in the post-liver transplant period [4]. Still, these studies have been mostly from registries, and there has been no data available from hospitalized patients.

We investigated national trends in liver transplant-related hospitalizations in the United States using the largest nationally representative database to address this knowledge gap. We also sought to explore clinic demographics, insurance information, and hospitalization costs in the context of hospital mortality.

## Materials and methods

Data source and study population

Data was collected from the Nationwide Inpatient Database (NIS) dated from 2007 to 2011. International Classification of Diseases, Ninth Revision Clinical Modification (ICD-9-CM) code 50.5 was used to identify all the liver transplant recipients. NIS is the most comprehensive publicly available all-payer database in the United States, funded by the Agency for Healthcare Research and Quality (AHRQ) as part of the Healthcare Cost and Utilization Project (HCUP). The database includes discharge-level data from around 1,000 hospitals, aiming to represent a 20% stratified sample of all community-based hospitals in the United States. The database consists of over a hundred clinical and nonclinical elements for each hospital visit, such as primary and secondary diagnoses and procedures, admission status, patient demographics, hospital characteristics, payer source, comorbidity measures, and DOH, among others.

Our study included the total number of 25,331 liver transplant recipients between 2007 and 2011. We sought to study the general trend in liver transplants, outcomes associated with the operations (in-hospital mortality, hospital charges, DOH), and independent factors associated with mortality in transplant recipients.

Statistical methods

Proportions in the respective percentages summarized the categorical variables. The Chi-square test compared the yearly trend. Continuous variables were summarized using means with standard error (SE) and t-test compared annual pattern. The multiple logistic regression models predicted independent factors associated with mortality in liver transplant recipients - a two-sided p-value of <0.05 assessed statistical significance. SAS 9.4 Software (SAS Institute Inc., Cary, North Carolina) performed the statistical analysis.

Our study was exempt from Institutional Review Board (IRB) review since it involves a publicly available de-identified database.

## Results

Demographics

**Figure 1:**
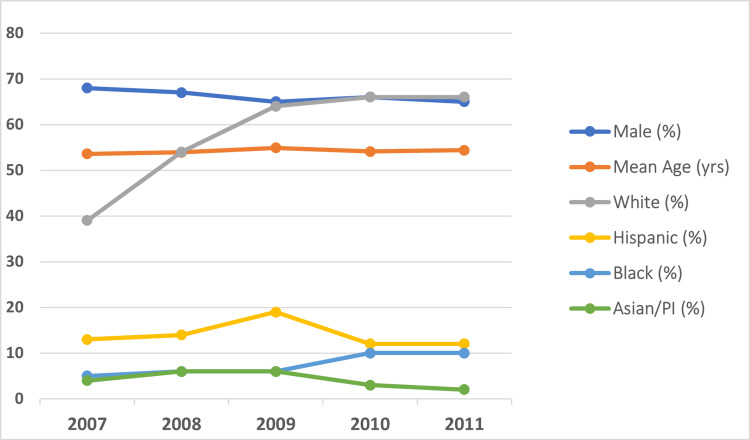
Trend in recipient demographics (age, sex, and race)

**Figure 2:**
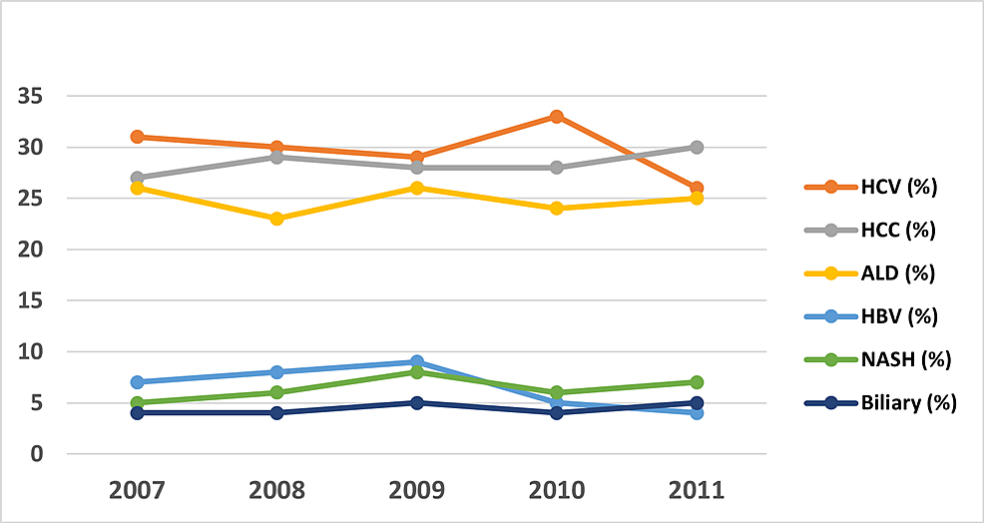
Trend in recipient characteristics as potential etiologies for liver transplant HCV - hepatitis C virus, HCC - hepatocellular carcinoma, ALD - alcoholic liver disease, HBV - hepatitis B virus, NASH - non-alcoholic steatohepatitis

The mean age of the transplant recipients was 54±0.19 years, with a majority of 51-65 years old, and 67% of the recipients were males (Table 1 and Figure 1). In terms of racial distribution, the majority were Caucasians (57%), followed by Hispanics (13%), African Americans (8%), and Asian/Pacific Islanders (4%). Most transplant recipients had a private healthcare plan (53%) and had average earnings in the upper quartile by zip code (26%). Patients with a higher Charlson Comorbidity Index (79% had a score of four) were more likely to be admitted to a southern hospital (33%), an academic hospital (>99%), and a large capacity hospital (90%). Seventy percent of liver transplant recipients received cadaver donors. Hepatitis C was the most prevalent reason for transplant (30%), followed by hepatocellular carcinoma (HCC) (29%), alcoholic liver disease (25%), hepatitis C, nonalcoholic steatohepatitis (NASH) cirrhosis (6%), and biliary cirrhosis (4%) (Figure 2).

**Table 1:** Demographics of liver transplant recipients NASH - non-alcoholic steatohepatitis, HCC - hepatocellular carcinoma

Covariates	Liver transplant population (N=25,331) %
Age	
Mean	54 years (0.19)
Median	55 years
Age category	
(18-35) years	6
(36-50) years	22
(51-65) years	62
≥66 years	10
Sex	
Male	67
Female	33
Race	
Caucasian	57
African American	8
Hispanic	13
Asian/Pacific Islander	4
Others/missing	18
Healthcare plan	
Medicare	28
Medicaid	13
Private	53
Self-pay	1
Others/missing	5
Medium household income by zip quartile	
Quartile 1	23
Quartile 2	24
Quartile 3	26
Quartile 4	25
Missing	2
Charlson Comorbidity Index (CCI)	
0-1	6
2	7
3	8
≥4	79
Mean	5.2 (0.1)
Hospital region	
Northeast	16
Midwest	25
South	33
West	26
Academic status of the hospital	
Non-academic	<1
Academic	>99
Capacity of the hospital	
Small	<1
Medium	9
Large	90
Transplant from cadaver donor	70
Potential causes for transplant	
Hepatitis C	30
HCC	29
Alcohol liver disease	25
Hepatitis B	6
NASH cirrhosis	6
Biliary cirrhosis	4

The trend in liver transplant outcomes

**Figure 3:**
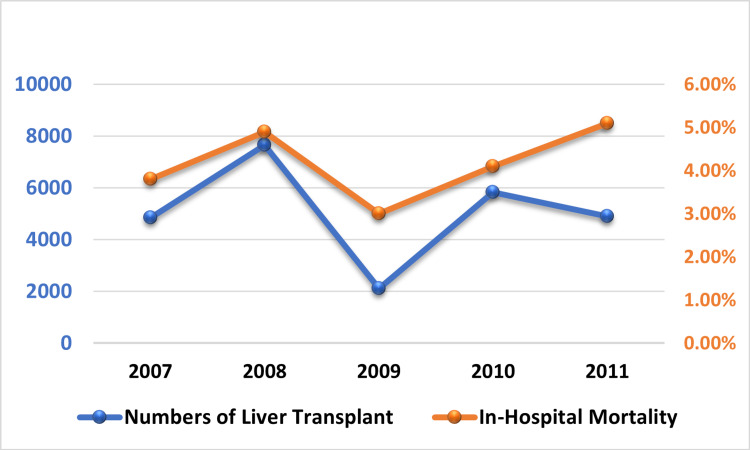
Trend in number of liver transplants and in-hospital mortality

The number of liver transplants increased from 4,844 to 4,902 by 1.2% from 2007 to 2011 (Table 2 and Figure 3). Mortality increased from 3.8% in 2007 to 5.1% in 2011, with no significant change in the yearly trend (p=0.5). There was a substantial increase in mean hospital charges from 2007 to 2011 (p=0.046 for the whole trend).

**Table 2:** Trend in the outcomes in liver transplant recipients DOH - duration of hospitalization *After excluding those who died in the hospital

Outcomes	2007 (N=4,844)	2008 (N=7,650)	2009 (N=2,114)	2010 (N=5,821)	2011 (N=4,902)	P-values for trend
Died, %	3.8%	4.9%	3.0%	4.1%	5.1%	0.5
Hospital charge (mean), $	335,504; 328,558*	353,324; 340,367*	386,283; 380,316*	399,901; 386,365*	498,369; 469,837*	0.046; 0.098*
DOH, days	17.4; 17.8*	21.6; 20.2*	20.7; 19.6*	19.9; 18.8*	22.7; 21.3*	0.11; 0.21*

**Figure 4:**
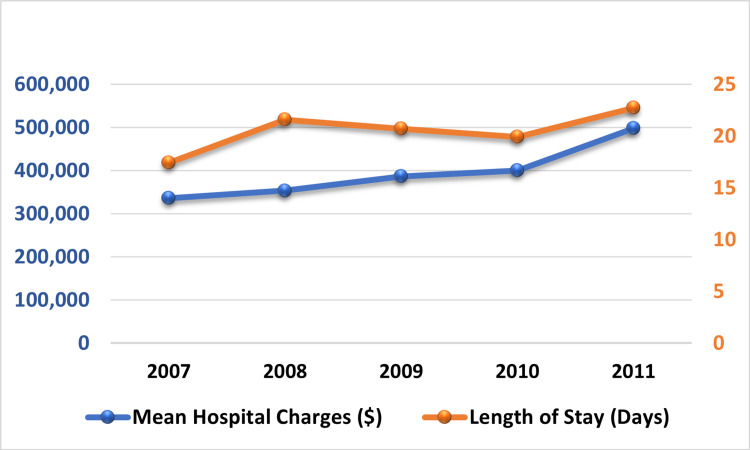
Trend in mean hospital charges and mean length of stay in liver transplant recipients

Still, there was no statistical difference in the yearly hospital charges except for those who died in the hospital (0.098) (Table 2 and Figure 4). Furthermore, there was no statistical difference in the yearly trend of the length of stay in the transplant recipients after both inclusion (p=0.11) and exclusion of those who died in the hospital (p=0.21).

Independent predictors of mortality

The independent factors affecting the mortality in liver transplant recipients were being African American (OR: 1.99, 95% CI: 1.25-3.18, p=0.005), admission in large capacity hospitals (OR: 2.54, 95% CI: 1.59-4.06, p<0.001), and liver donation via cadaver (OR: 0.65, 95% CI: 0.50-0.84, p=0.002; Table 3). Independent factors protective of mortality were having a private healthcare plan (OR: 0.70, 95% CI: 0.51-0.97, p=0.03), being admitted in an academic hospital (OR: 0.55, 95% CI: 0.37-0.83, p=0.005), having a diagnosis of HCC (OR: 0.59, 95% CI: 0.40-0.87, p=0.009) and having a diagnosis of NASH cirrhosis (OR: 0.38, 95% CI: 0.17-0.86, p=0.02).

**Table 3:** Independent predictors of in-hospital mortality in liver transplant recipients CI - confidence interval, NASH - non-alcoholic steatohepatitis

Predictors	Odds ratio	Lower limit of 95% CI	Upper limit of 95% CI	P
Age category				
(18-35) years	Ref	Ref	Ref	Ref
(36-50) years	0.850	0.549	1.316	0.4595
(51-65) years	1.025	0.621	1.693	0.9218
≥66 years	1.162	0.572	2.361	0.6735
Sex				
Female	Ref	Ref	Ref	Ref
Male	0.757	0.551	1.038	0.0828
Race				
White	Ref	Ref	Ref	Ref
Black	1.993	1.249	3.179	0.0046
Hispanic	1.452	0.947	2.225	0.0858
Asian/Pacific-Islander	0.764	0.369	1.581	0.4609
Others/missing	1.083	0.669	1.753	0.7416
Insurance status				
Medicare	Ref	Ref	Ref	Ref
Medicaid	1.014	0.668	1.537	0.9484
Private	0.699	0.506	0.965	0.0303
Self-pay	1.384	0.518	3.701	0.5102
Others/missing	0.848	0.411	1.752	0.6509
Income quartile by zip code				
Quartile 1 (poorest)	Ref	Ref	Ref	Ref
Quartile 2	1.225	0.828	1.812	0.3041
Quartile 3	0.909	0.615	1.344	0.6262
Quartile 4 (richest)	0.860	0.543	1.363	0.5151
Charlson Comorbidity Index (CCI)				
0-1	Ref	Ref	Ref	Ref
2	0.690	0.277	1.719	0.4190
3	1.097	0.468	2.572	0.8282
≥4	0.985	0.517	1.876	0.9631
Hospital region				
Northeast	Ref	Ref	Ref	Ref
Midwest	0.616	0.321	1.182	0.1419
South	0.745	0.430	1.292	0.2885
West	0.798	0.400	1.594	0.5167
Teaching status of the hospital				
Non-teaching	Ref	Ref	Ref	Ref
Teaching	0.554	0.370	0.829	0.0048
Hospital bed size				
Small bed size	Ref	Ref	Ref	Ref
Medium bed size	0.703	0.181	2.725	0.6044
Large bed size	2.537	1.587	4.057	0.0002
Donor characteristics				
Non-cadaver donor	Ref	Ref	Ref	Ref
Cadaver donor	0.646	0.496	0.842	0.0017
Potential etiologies of liver transplant				
Hepatocellular carcinoma	0.592	0.402	0.872	0.0089
Hepatitis C virus	0.719	0.487	1.060	0.0941
Hepatitis B Virus	0.789	0.453	1.374	0.3957
Alcoholic liver disease	0.846	0.613	1.168	0.3029
Biliary cirrhosis	0.724	0.320	1.635	0.4298
NASH cirrhosis	0.383	0.171	0.860	0.0209

## Discussion

In this study, we present a vital observation of increasing mortality trends despite advances in liver transplantation treatment modalities since 2007. Our study results were similar to the survey conducted by Stepanova et al. [3]. They have also shown that African American patients have a lower five-year survival rate than other races [4,5]. The above studies have implicated differences in socioeconomic status, insurance type, and educational background as a possible explanation for worse outcomes in the African American population [4-6]. African American race and hepatitis C were also independent predictors of mortality in UNOS/ Organ Procurement and Transplantation Network (OPTN) registry studies [7-9].

Our findings were of significance and contrast to the retrospective study conducted by Lee et al. in pre-liver transplant patients suggesting no significant racial/ethnic differences in post-liver transplant survival before the year 2000 [10]. According to our findings, African American patients have an increased risk of mortality post-liver transplantation. Our study also suggested that private insurance results in lower mortality, which can indirectly reflect patients' socioeconomic status. We also noted large capacity hospitals as an independent predictor of higher mortality in the post-liver transplant period. There have been conflicting studies about transplant center volume, which is proportional to hospital capacity and survival outcome [11].

The majority of studies identified an inverse relationship in terms of high volume and mortality [11,12]. In contrast, few studies did not show a significant relation between transplant center volume and mortality outcomes [13]. High volume transplant centers tend to take sicker patients with high MELD scores and possible excess postoperative complications, including mortality. Low volume transplant centers tend to have more stringent liver selection criteria for liver transplant listing due to their limited resources in handling postoperative care.

There were several independent factors associated with decreased mortality in our observational study. Various other studies have shown a negative impact on survival outcomes in patients with multiple chronic medical conditions such as type 2 diabetes mellitus [3,9]. Patients with private insurance had decreased mortality compared to Medicare patients. DuBay et al., in their retrospective study involving the Scientific Registry of Transplant Recipients, made similar observations [14]. Few studies addressed insurance status and cost barriers, especially for cancer care [15]. Glueckert et al. identified the most favorable outcomes with private insurance regarding rejection episodes, missed clinic appointments, and hospital readmission rates compared to the charity care population. Glueckert et al. also inferred that transportation issues in post-liver transplant care as a possible explanation for discrepancy across insurance cohorts [16].

There are several limitations of our study which are inherent to the National Inpatient Sample (NIS) database. For example, NIS does not include outpatient data and those who died before hospitalization. Although the NIS database utilizes a sampling mechanism, there is a possibility of the existence of bias. NIS database can not track longitudinal follow-ups of a single individual with multiple hospitalizations. As in other observational studies, there is a possibility of unmeasured confounders.

## Conclusions

Our study found an increasing trend in worse outcomes (increased mortality, average hospital costs, and average DOH) after a liver transplant. Patients of the African American race and large capacity hospitals were associated with a higher risk of death, whereas private healthcare plans, academic hospitals, cadaver donors, HCC, and NASH cirrhosis were associated with a lower risk. Further evaluations from future clinical trials and intervention studies are needed to assess the mortality trend in recent years given the upcoming improvements in chemotherapy, including immunotherapy modalities.
